# Matrix metalloproteinase 2 level in human follicular fluid is a reliable marker of human oocyte maturation in *in vitro fertilization* and *intracytoplasmic sperm injection* cycles

**DOI:** 10.1186/s12958-015-0099-8

**Published:** 2015-09-04

**Authors:** Wen-Jui Yang, Fon-Chang Liu, Jih-Sheng Hsieh, Ching-Hung Chen, Shun-Yu Hsiao, Chih-Sheng Lin

**Affiliations:** Department of Biological Science and Technology, National Chiao Tung University, Hsinchu City, Taiwan; Department of Fertility and Reproductive Medicine, Ton-Yen General Hospital, Hsinchu County, Taiwan; Department of Pharmacy, Wei Gong Memorial Hospital, Miaoli County, Taiwan; Department of Surgery, Mackay Memorial Hospital, Hsin-Chu Branch, No.690, Sec. 2, Guangfu Road, Hsinchu City, 30071 Taiwan; Division of Infertility and Reproductive Medicine, Taiwan IVF Group Center, Hsinchu City, Taiwan

**Keywords:** Follicular fluid, *in vitro* fertilization, Matrix metalloproteinase, Oocytes maturation, Tissue inhibitor of matrix metalloproteinase

## Abstract

**Background:**

To determine whether matrix metalloproteinases (MMPs) and their tissue inhibitors (TIMP-1 and TIMP-2) in human follicular fluid, have any relationships with oocyte maturation *in vivo* and subsequent fertilization during *in vitro fertilization* (IVF) or *intracytoplasmic sperm injection* (ICSI) cycles.

**Methods:**

The follicular fluids were obtained from 150 female patients undergoing IVF/ICSI cycles and a total of 1504 oocytes were retrieved for analysis. MMP-2 and MMP-9 activities were measured using zymography assay. TIMP-1 and TIMP-2 concentrations were quantitatively assessed using enzyme-linked immunosorbent assay (ELISA).

**Results:**

Human follicular fluid MMP-2 level was significantly associated with the rate of maturity of oocytes (*P* < 0.001). Furthermore, the MMP-2 was significantly associated with the higher fertilization rate (*P* < 0.01). There was no significant correlation between follicular MMP-9 and the maturation rate of oocytes. The TIMP-1 and TIMP-2 also showed no correlation with the oocyte maturation rate.

**Conclusions:**

The level of gelatinase MMP-2 in human follicular fluid might be a reliable marker of mature oocytes during IVF/ICSI cycles. Furthermore, the MMP-2 expression has a strong association with higher fertilization rate. Further studies are needed to support this theory.

## Background

During an assisted reproductive technology cycle, human pregnancy is dependent on a number of physiologic conditions, including oocyte maturation, successful fertilization, and embryonic blastocyst development [1, 2]. Currently, the clinical prediction of oocyte maturation in *in vitro fertilization* (IVF) / *intracytoplasmic sperm injection* (ICSI) cycles mainly depend on the size of leading follicles (>17 mm in diameter) and are sometimes accompanied by serum estradiol level. However, there is still no accurate marker for the prediction of the maturation of oocytes. Recently, matrix metalloproteinases (MMPs) have been shown to be important in the follicular microenvironment, as well as subsequent ovulation [3–5]. MMPs are a family of zinc endopeptidases capable of degrading all of the components of the extracellular matrix (ECM) and are divided into sub-groups depending on the specificity of the substrates [6]. Matrix metalloproteinase-2 (MMP-2) and matrix metalloproteinase-9 (MMP-9) belong to gelatinases and their activities are specifically inhibited by tissue inhibitors of metalloproteinases (TIMPs). Tissue inhibitor of metalloproteinase-1 (TIMP-1) has a higher affinity for MMP-9, while tissue inhibitor of metalloproteinase-2 (TIMP-2) has a higher affinity for MMP-2 [6–8]. In addition, MMP and TIMP proteins have been detected in the sperm-oocyte interaction. This finding might suggest that these proteins have the potential for oocyte maturation and subsequent fertilization [9].

The production of MMPs and TIMPs as well as the mechanisms of action in the follicular microenvironment are important for subsequent follicular development. Deficient follicular growth and/or ovulation are correlated with the presence of low levels of MMPs in follicular fluid [3, 4]. Gelatinases have an important role in the stabilization of the ECM, an important process during the initiation of pregnancy [5, 6]. In IVF/ICSI cycles, it is well-known that not all oocytes retrieved after controlled ovarian hyper-stimulation show the same potential for achieving maturity. Between 5 and 20 % of retrieved oocytes are immature and have a low fertilization rate [10]. Therefore, the accurate marker of the maturity rate of retrieved oocytes is important for the success of assisted reproductive technologies. We wondered whether the expression of MMPs (MMP-2 and MMP-9) and TIMPs (TIMP-1 and TIMP-2) in follicular fluid during the IVF/ICSI cycle is related to the maturity of oocytes and sought to find whether MMPs in follicular fluid are a reliable marker for predicting the maturation rate of oocytes.

## Methods

### Subject selection

This prospective study included IVF/ICSI cycles during the period from 2010–2013 in the Fertility Unit of Ton-Yen General Hospital, Taiwan. To minimize confounding factors, patients diagnosed with polycystic ovaries and a poor response (< two retrieved oocytes and serum estradiol [E_2_] level ≤ 300 pg/ml on the day of human chorionic gonadotrophin [hCG] administration) were excluded from the study. Patients with male factor infertility (which was defined by the presence of any of the following parameters: sperm concentration < 20 × 10^6^/ml; total motility < 40 %; and normal morphology < 4 %) were treated with ICSI, while others were treated with conventional insemination for IVF. The study was performed with the approval of the Institutional Review Board of Ton-Yen General Hospital. All participants provided written informed consent to participate in this study.

### Ovarian stimulation

One hundred and fifty patients who underwent IVF/ICSI were included in this study. Briefly, all of the patients used a GnRH antagonist protocol. Recombinant follicle stimulating hormone (Gonal-F; Serono Laboratories, Aubonne, Switzerland) and human menopausal gonadotropin (Menopur; Ferring GmbH, Kiel, Germany) were administered daily, beginning on the second day of the menstrual cycle. The doses were adjusted according to the patient’s individual ovarian response. When the dominant follicle reached a mean diameter of 12 mm, cetrorelix (Cetrotide; Serono Laboratories, Baxter Oncology GmbH, Halle, Germany) was administered subcutaneously at a dose of 0.25 mg daily until the day of human chorionic gonadotropin (hCG; Serono Laboratories) administration.

Ovulation was induced with 10,000 IU of hCG when the patients had ≥ two follicles with diameter > 17 mm. Oocytes were retrieved 34–36 h after hCG administration under guided vaginal sonography and exposed to spermatozoa for insemination. Upon completion of oocyte collection and IVF/ICSI, embryos were graded morphologically by two senior embryologists.

Oocytes exhibiting two pronuclei (2PN) and two polar bodies 16-20 h after insemination/ICSI were further incubated for embryonic development.

Those displaying two pronuclei were sequentially cultured further in groups up to the blastocyst stage (Day 5) in a humidified atmosphere containing 5 % O_2_ and 6 % CO_2_. Blastocyst quality was defined according to the criteria presented by Gardner and Schoolcraft [11] and briefly described as follows: Blastocysts were graded from 1 to 6 based on their degree of expansion and hatching status (from blastocoeles less than half of the volume of the embryo (grade 1) to hatched blastocyst (grade 6)). For blastocysts of grades 3 to 6, inner cell mass (ICM) and trophectoderm (TE) were also evaluated and graded accordingly. The ICM was graded into three categories: A (many ICM cells packed together tightly), B (several ICM cells grouped loosely) and C (very few ICM cells). The trophectoderm was also graded into three categories: A (many trophectoderm cells forming a multiple epithelium layer), B (few trophectoderm cells consisting of a loose epithelium layer) and C (very few trophectoderm cells). In this study, we defined good-quality blastocysts as ≥ 3AA; 3, 4, 5, 6, AB, BA, BB and AC. We defined poor-quality blastocysts as ≤ 3BB; 3, 4, 5, 6 and CA. Additionally, we defined the other two groups based on the rate of blastocyst formation and quality: the high day 5 good blastocyst group (high day 5 GB group) (day 5 embryonic blastocyst rate ≥ 50 % and high-quality blastocyst rate ≥ 50 %) and the low day 5 good blastocyst group (low day 5 GB group) (day 5 embryonic blastocyst rate < 50 % and high-quality blastocyst rate < 50 %). According to these two groups, we calculated the oocyte maturation rate and the oocyte fertilization rate.

### Collection of ovary follicular fluid

The ovary follicular fluid was collected following the procedures previously described by Rosen et al. [12] and Lee et al. [13]. Each follicle was pierced with a single lumen needle and completely aspirated by ultrasound-guided aspiration. Follicular fluids of each patient were pooled. After oocyte removal, follicular fluid samples were centrifuged (300 × *g* for 10 min) to remove blood and granulosa cells. Finally, the supernatant was aliquoted into 2 ml cryovials and stored at −80 °C until assays could be performed.

### Zymography for the assay of gelatinolytic activity

The activities of gelatinases (MMP-2 and MMP-9) in follicular fluid were carried out using zymography, as previously described by our laboratory [14, 15]. Briefly, follicular fluid samples were analyzed by the gels containing gelatin (0.1 % w/v). Each lane of zymographic gel was loaded with a constant volume of the samples of follicular fluid. After electrophoresis, gels were incubated overnight at 37 °C in Tris buffer (pH 8) with 5 mM CaCl_2_. Finally, the gels were stained with Coomassie blue, and the gelatinolytic activities were presented as transparent bands on the blue background. Gelatinolytic activities were identified as clear zones and calculated densitometric value of the lyses against a dark blue background on zymography gels using ImageJ software (National Institutes of Health, Bethesda, MD, USA), which quantified both the surface and the intensity of the lysis bands after scanning of the gels according to a previous report by Hu and Beeton [16]. The levels of MMP-2 and MMP-9 gelatinolytic activity are presented as arbitrary units representing the densitometric concentrations after standardization with levels of recombinant MMP-2 (#M9070; Sigma-Aldrich, St. Louis, MO, USA) and MMP-9 (#M8945; Sigma-Aldrich) as controls in the same gel, respectively, according to the study of Lee et al. [13] and Sessions et al. [17].

**Fig. 1:**
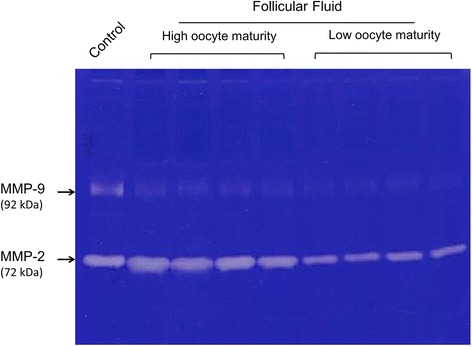
Representative MMP-2 and MMP-9 zymography gel of human follicular fluids. Zymography assay was performed as described in the section of Materials and Methods. The gel was analyzed by 8 % SDS-PAGE and followed by Coomassie staining. There was related higher MMP-2 activity compared with MMP-9 activity detected in the follicular fluids. The zymography gel was loaded with 5 μl of samples diluted 5× in PBS to quantify proMMP-2 and proMMP-9 activity. The representative gel shows follicular fluids from 4 patients with 100 % MII oocytes (high oocyte maturity) and 4 patients with <100 % MII oocytes (low oocyte maturity), as well as one control lane with recombinant proMMP-2 and proMMP-9. MMP-9 (92 kDa) and MMP-2 (72 kDa) on the zymography gel were indicated

Representative MMP zymography assay of ovary follicular fluid was shown in Fig. 1. Almost of MMP-2 and MMP-9 detected in the sample of follicular fluids is latent form in the present study. Therefore, in this study, we could only detect and discuss the latent form of MMP-2 and MMP-9.

### Enzyme-linked immunosorbent assay (ELISA) for TIMPs

The concentrations of TIMPs in follicular fluid were determined using human TIMP-1 and TIMP-2 ELISA kits (Abcam, Cambridge, MA, USA) according to the manufacturer’s recommendations. The samples were diluted appropriately to fall within the standard range of the assay. Each recombinant human TIMP-1 and TIMP-2 provided in the ELISA kits was used as a standard. The diluted follicular fluid was incubated in ELISA plates in which wells had been coated with anti-human TIMP-1 and TIMP-2 primary antibodies. Following the addition of biotinylated secondary antibodies, the plates were washed and reacted with horseradish peroxidase (HRP)-conjugated streptavidin. Tetramethylbenzidine (TMB) one-step substrate was used to detect the targeted protein and the product was measured at 450 nm using a micro-plate reader (Thermo Scientific Multiskan EX, Waltham, MA, USA).

### Statistical analysis

The data were analyzed using SPSS (version 19.0 for Windows; SPSS, Chicago, IL, USA). All parameters are presented as the mean with the standard deviation (SD). Pearson’s test was used for correlation analysis between variables. Significant differences between the subgroups were evaluated using one-way analysis of variance (ANOVA). A *P* value of < 0.05 was considered statistically significant.

## Results

In the present study, 150 women who underwent IVF/ICSI cycles were analyzed. The patients’ characteristics are shown in Table 1. A total of 1504 oocytes were retrieved and 1346 of them were metaphase 2 (MII) oocytes. The mean number of MII oocytes per patient was 9.1 ± 6.2. The MII oocyte ratio was 91.3 %. Approximately 956 embryos were normally fertilized (i.e., 2PN embryos) and 430 of them developed good-quality blastocysts at day 5 (i.e., D5 GB embryos) (Table 1). The 2PN embryo ratio and the D5 GB embryo ratio were 71.4 and 35.6 %, respectively.

**Table 1:** Characteristic measurements of IVF/ICSI women

Number of patients	150
Age (years)	35.2 ± 4.4
Serum estradiol (pg/ml)	2692 ± 1589
Serum progesterone (ng/ml)	0.73 ± 0.54
Mean number of retrieved oocytes	10.2 ± 6.8 (1504)
Mean number of MII oocytes	9.1 ± 6.2 (1346)
MII oocyte ratio (%)	91.3 ± 14.9 (1346/1504)
Mean number of 2PN embryos	6.5 ± 4.8 (956)
2PN embryo ratio (%)	71.4 ± 24.2 (956/1346)
Mean number of D5 GB embryos	3.0 ± 3.9 (430)
D5 GB embryo ratio (%)	35.6 ± 33.9 (430/956)
MMPs in follicular fluid	
MMP-2 (Arbitrary units/μl)	18.4 ± 4.51
MMP-9 (Arbitrary units/μl)	2.13 ± 6.49
TIMPs in follicular fluid	
TIMP-1 (ng/ml)	117 ± 35
TIMP-2 (ng/ml)	54.5 ± 15.5

*MII* Metaphase II, *2PN* Two pronuclei, *D5 GB* good-quality blastocysts on day 5 *MMP* Matrix metalloproteinase, *TIMP* Tissue inhibitor of matrix metalloproteinase

Values are expressed as the mean ± SD. The values in parentheses indicate the numbers of oocytes or embryos

### MMPs and TIMPs in follicular fluid

In the follicular fluid samples from all women, the MMP-2 and MMP-9 activities were 18.4 ± 4.51 and 2.13 ± 6.49 (arbitrary units/μl), respectively. The TIMP-1 and TIMP-2 levels were 117 ± 35 and 54.5 ± 15.5 ng/ml, respectively (Table 1). To evaluate the relationships between serum E2 and progesterone concentrations, MMP activity, TIMP level, maturation rate, fertilization rate of oocytes and day 5 good-quality blastocyst rate, the Pearson correlation coefficient was analyzed. The data indicated that MII% are significantly and positively correlated to follicular MMP-2 activity (r = 0.261; *P* < 0.01). However, the day 5 good-quality blastocyst rate had no significant correlation to the activity of follicular MMPs and TIMPs (data not shown). According to these results, we proposed that the MMP-2 is a reliable factor associated with oocyte maturation in IVF/ICSI cycles.

### Association of oocyte maturation rate with MMP-2

To further investigate the relationship between gelatinases and the high as well as low maturity rate of oocytes retrieved, the patients were divided into two groups: the high oocyte maturity group (MII ratio = 100 %; *n* = 95) and the low oocyte maturity group (MII ratio < 100 %; *n* = 55) (Table 2). In our clinic, only follicles ≥ 16 mm were retrieved. More than half of our cases had an oocyte maturation rate = 100 % (*n* = 95). To avoid statistical bias, we defined the cut-point as an oocyte maturation rate =100 %. The results show that there were no differences between two groups in serum E2 level, serum progesterone level, 2PN embryo ratio, and follicular fluid level of MMP-9, TIMP-1 and TIMP-2; however, the activity of MMP-2 in follicular fluid was increased in the high maturity oocyte group (19.6 ± 4.3 *vs.*16.3 ± 4.3 (arbitrary units/μl), *P* < 0.001) (Table 2). The D5 GB ratio was significantly higher in the high maturity oocyte group (45.8 ± 32.5 *vs.* 29.8 ± 33.5, *P* < 0.01).

**Table 2:** MMP and TIMP activities in the follicular fluid between high and low oocyte maturity groups

	High oocyte maturity group (MII oocytes = 100 %)	Low oocyte maturity group (MII oocytes < 100 %)	*P* value
Number of patients	95	55	
Estradiol (pg/ml)	2774 ± 1078	2651 ± 1829	NS
Progesterone (ng/ml)	0.81 ± 0.78	0.69 ± 0.39	NS
MII oocytes ratio (%)	100 ± 0	76.4 ± 15.8	< 0.001
2PN embryos ratio (%)	75.9 ± 24.4	68.8 ± 23.8	NS
D5 GB embryos ratio (%)	45.8 ± 32.5	29.8 ± 33.5	< 0.01
MMP-2 (Arbitrary units/μl)	19.6 ± 4.3	16.3 ± 4.3	< 0.001
MMP-9 (Arbitrary units/μl)	2.11 ± 0.59	2.19 ± 0.74	NS
TIMP-1 (ng/ml)	123 ± 37	107 ± 26	NS
TIMP-2 (ng/ml)	54.6 ± 16.5	52.6 ± 13.6	NS

*MII* Metaphase II, *2PN* Two pronuclei, D*5 GB* Good-quality blastocysts on day 5, *MMP* Matrix metalloproteinase, *TIMP* Tissue inhibitor of matrix metalloproteinase

Values are expressed as the mean ± SD. *P* values are comparisons between the high- and low-maturity groups by ANOVA.; NS: no statistical significance (i.e., *P* > 0.05)

### Association of formation rate of two pronuclei (2PN) with MMPs and TIMPs

To investigate the relationships between gelatinase activity and the oocyte fertilization, we divided patients into another two groups: the low fertilization rate group (2PN rate < 70 %; *n* = 84) and the high fertilization group (2PN rate ≥ 70 %; *n* = 66) (Table 3). There were no significant differences in the serum level of E2, the serum level of progesterone, MMP-9, TIMP-1 and TIMP-2 in follicular fluid between two groups. There was a significant difference in follicular fluid MMP-2 level between the high and low fertilization rate groups (19.4 ± 4.2 *vs.* 17.5 ± 4.6 (arbitrary units/μl), *P* < 0.01) (Table 3). The MII oocyte ratio was significantly higher in the high fertility rate group (95.2 ± 11.9 *vs.* 88.3 ± 16.3, *P* < 0.01) and the 2PN embryo ratio was significantly elevated in the high fertility rate group (88.5 ± 11.0 *vs.* 49.6 ± 18.1, *P* < 0.001). The D5 GB embryo ratio was also higher in the high fertility rate group (42.8 ± 33.4 *vs.*26.6 ± 32.5, *P* < 0.01).

**Table 3:** MMP and TIMP activities in the follicular fluid between the high and low percentage successful fertilization groups

	High fertility rate group (2PN ≥ 70 %)	Low fertility rate group (2PN < 70 %)	*P* value
Number of patients	84	66	
Estradiol (pg/ml)	2689 ± 1588	2707 ± 1611	NS
Progesterone (ng/ml)	0.72 ± 0.49	0.74 ± 0.60	NS
MII oocytes ratio (%)	95.2 ± 11.9	88.3 ± 16.3	< 0.01
2PN embryos ratio (%)	88.5 ± 11.0	49.6 ± 18.1	< 0.001
D5 GB embryos ratio (%)	42.8 ± 33.4	26.6 ± 32.5	< 0.01
MMP-2 (Arbitrary units/μl)	19.4 ± 4.2	17.5 ± 4.6	< 0.01
MMP-9 (Arbitrary units/μl)	2.14 ± 0.70	2.10 ± 0.59	NS
TIMP-1 (ng/ml)	117 ± 32	120 ± 35	NS
TIMP-2 (ng/ml)	55.1 ± 15.1	53.2 ± 17.0	NS

*MII* Metaphase II, *2PN* Two pronuclei, *D5 GB* Good-quality blastocysts on day 5, *MMP* Matrix metalloproteinase, *TIMP* Tissue inhibitor of matrix metalloproteinase

Values are expressed as the mean ± SD. *P* values are comparisons between the high and low percentage successful fertilization groups by ANOVA. NS: no statistical significance (i.e., *P* > 0.05)

### Association of good quality blastocysts at day 5 (D5 GB) with MMPs and TIMPs

To investigate the relationships between gelatinase activity and the day 5 good quality blastocyst formation rate, we divided patients to two groups: the high day 5 good blastocyst group (high day 5 GB group) (day 5 embryonic blastocyst rate ≥ 50 % and high quality blastocyst rate ≥ 50 %; *n* = 59) and low day 5 good blastocyst group (low day 5 GB group) (day 5 embryonic blastocyst rate < 50 % and high quality blastocyst rate < 50 %; *n* = 91). There were no significant differences in the serum level of estradiol, progesterone, MII oocytes ratio, follicular fluid level of MMP-2, MMP-9, TIMP-1, and TIMP-2 (Table 4).

**Table 4:** MMP and TIMP activities in the follicular fluid between high and low D5 GB embryo groups

	High D5 GB group (D5 GB ≥ 50 %)	Low D5 GB group (D5 GB < 50 %)	*P* value
Number of patients	59	91	
Estradiol (pg/ml)	2937 ± 1362	2541 ± 1715	NS
Progesterone (ng/ml)	0.83 ± 0.76	0.68 ± 0.36	NS
MII oocytes ratio (%)	89.7 ± 14.6	92.4 ± 15.0	NS
2PN embryos ratio (%)	77.0 ± 21.0	67.7 ± 25.5	< 0.05
D5 GB embryos ratio (%)	72.4 ± 16.3	11.8 ± 16.5	< 0.001
MMP-2 (Arbitrary units/μl)	17.2 ± 4.3	19.1 ± 4.5	NS
MMP-9 (Arbitrary units/μl)	2.25 ± 0.66	2.04 ± 0.63	NS
TIMP-1 (ng/ml)	114 ± 30	120 ± 36	NS
TIMP-2 (ng/ml)	54.9 ± 14.8	54.3 ± 16.1	NS

*MII* Metaphase II, *2PN* Two pronuclei, *D5 GB* Good-quality blastocysts on day 5, *MMP* Matrix metalloproteinase, *TIMP* Tissue inhibitor of matrix metalloproteinase

Values are expressed as the means ± SD. *P* values are comparisons between the high and low D5 GB embryo groups by ANOVA.; NS: no statistical significance (i.e., *P* > 0.05)

## Discussion

In this study, we showed that the follicular fluid MMP-2 level during IVF/ICSI treatment had a significant correlation with the oocyte maturation rate (Table 2). In addition, there was no correlation between the oocyte maturation rate with MMP-9, TIMP-1 and TIMP-2 in follicular fluid. Furthermore, we suggested that the MMP-2 level in follicular fluid was significantly higher in the high fertility rate group (2PN ≥ 70 %) (Table 3). The accurate volume of follicular fluids was hard to obtain, therefore a constant volume was used instead in each patient. There are more fluids expected in larger follicles, which contain mature oocytes. The diluting effect in larger follicles would have presumably resulted in less proMMP-2 activity, which was not observed in our study (more activity was measured instead), suggesting an increased production of proMMP-2 in mature follicles.

To the best of our knowledge, the present study was the first study to find human follicular fluid MMP-2 level could predict oocyte maturation in IVF/ICSI cycles. In IVF/ICSI treatment, oocyte maturation based on the size of leading follicles (> 17 mm) and combined with serum E2 level (assumed elevation level of serum E2 is approximately 150–200 ng/dl per mature oocyte) could be useful in the clinical prediction of oocyte maturation. However, these predictions were not always accurate. In the present study, we showed that the follicular fluid MMP-2 level was not only significantly correlated to the maturation rate of oocytes but also strongly correlated to the normal fertilization rate. In addition, the serum E2 level was not significantly correlated to the oocyte maturity rate and fertility rate (Tables 2 and 3). These data indicate that MMP-2 might play a major role in oocyte maturation and further fertilization.

According to the study of Brew et al. [8], it was reported that the MMP-TIMP system is implicated in the proteolysis network of follicular development and the breakdown of the follicular wall during successful ovulation. In an *in vitro* culture study involving bovine follicles, the secretion of MMP-9, TIMP-1 and TIMP-2 was shown to be associated with follicular health [18]. The IVF/ICSI patients with higher levels of MMPs, especially MMP-9, in the follicular fluid and serum at the time of oocyte retrieval, had higher pregnancy rates after embryo transfer [13, 19]. The levels of MMP expression and activity during follicular development, however, have been reported inconsistently. Compared with MMPs in the follicular fluid of IVF/ICSI patients and normal ovulating women, the MMP-2 and MMP-9 levels were much lower in IVF/ICSI patients [3]. Recently, some evidence has shown that MMP-2 may play a role in follicular development and MMP-9 might be involved in follicular breakdown [4]. Compared with MMPs, the changes in the follicular TIMPs related to oocyte maturation and fertilization have received limited attention. In infertile patients, an increase in the follicular TIMP-1, but not TIMP-2, level was detected compared with that in the normally ovulating control group. According to the results, multiple and important functions of ovarian TIMP-1 during the folliculogenesis and ovulation process were proposed [20, 21]. However, reports of significant differences between the groups with altered performance in infertile patients are lacking, such as oocyte *in vitro* maturation and fertilization. The previous study showed that MMP expression was elevated while TIMP expression was reduced at the time of ovulation [22]; others had concluded that the high level of TIMPs was unchanged during follicular development [23]. In the present study, we found that the concentrations of TIMP levels, including TIMP-1 and TIMP-2, in follicular fluid were not related to the oocyte maturation rate (Table 2). Furthermore, the TIMPs were not correlated to the normal fertilization rate of embryos (Table 3). These findings were compatible with the previous study [2].

In controlled ovarian hyper-stimulation cycles, evidence showed epidermal growth factor receptor (EGFR) signaling as a key factor to coordinate LH-mediated events [24–26]. Furthermore, this EGFR activation was required for MMP-mediated release of membrane-bound EGFR ligands [27, 28]. Similar effects of EGFR phosphorylation and gonadotropin-induced oocyte maturation were shown to be diminished after inhibiting MMP activity in ovarian follicles [24, 25, 29]. It had also been reported that an increase in bovine MMPs was clearly associated with subsequent blastocyst development during *in vitro* maturation [30]. In the previous studies, the inhibition of MMP-2 activity significantly decreased the fertilization rate [31, 32]. Compatible with previous studies, we found that MMP-2 activity was significantly correlated to the maturation rate of oocytes (Table 2). According to the findings above, we proposed that MMP-2 might play a crucial role during oocyte maturation.

Mammalian fertilization requires the penetration of the sperm through the zona pellucida of oocytes. Defective sperm-zona pellucida interaction, including hardening of the zona pellucida, may cause fertilization failure. Therefore, to further penetrate the zona pellucida for fertilization is important, especially in IVF treatment [33]. Several proteolysis regulators, including MMP-2, TIMP-1, TIMP-2 and TIMP-3, have been detected in seminal fluid and are important for mammalian sperm function in fertilization [9]. In addition, female-expressed proteolysis regulators are also essential, as they may act together with male-derived proteases and protease inhibitors in post-mating proteolysis networks. In the previous study, MMP-2 activity was shown to associate with the inner acrosomal membrane of sperm; the proteolytic activity might improve sperm penetration in the zona pellucida [31]. In our study, MMP-2 activity in the follicular fluid of women undergoing IVF/ICSI showed a significant surge in the high oocyte maturity and high fertilization rate subgroups. It was reasonable to think that when oocytes were going to mature, the MMP-2 showed advanced activity in the follicular fluid to prepare for further fertilization. If the follicular fluid MMP-2 level was a marker of oocyte maturation, then serum MMP-2 level might be a reliable marker as well. Further studies on serum MMP-2 levels can be performed to prove this hypothesis. This finding further suggests that MMP-2 might be an important enzyme to improve the function of matured oocytes during the oocyte-sperm interaction and increase the successful rate of fertilization during IVF/ICSI.

In conclusion, MMP-2 level in follicular fluid during IVF/ICSI was significantly correlated with the maturation rate of oocytes. Furthermore, MMP-2 activity also had a strong correlation with the higher fertilization rate. MMP-2 activity in follicular fluid in IVF/ICSI cycles may be a reliable marker of the oocyte maturation rate. However, further studies are warranted to confirm these findings.

## References

[1] Sakkas D, Percival G, D’Arcy Y, Sharif K, Afnan M (2001). Assessment of early cleaving in vitro fertilised human embryos at the 2-cell stage before transfer improves embryo selection. Fertil Steril.

[2] Geary TW, Smith MF, MacNeil MD, Day ML, Bridges GA, Perry GA (2013). Triennial Reproduction Symposium: influence of follicular characteristics at ovulation on early embryonic survival. J Anim Sci.

[3] D’Ascenzo S, Giusti I, Millimaggi D, Marci R, Tatone C, Cardigno-Colonna R (2004). Intrafollicular expression of matrix metalloproteinases and their inhibitors in normally ovulating women compared with patients undergoing in vitro fertilization treatment. Eur J Endocrinol.

[4] Goldman S, Shalev E (2004). MMPS and TIMPS in ovarian physiology and pathophysiology. Front Biosci.

[5] Stamouli A, O’Sullivan MJ, Frankel S, Thomas EJ, Richardson MC (1996). Suppression of matrix metalloproteinase production by hCG in cultures of human luteinized granulosa cells as a model for gonadotropin-induced luteal rescue. J Reprod Fertil.

[6] Gaide Chevronnay HP, Selvais C, Emonard H, Galant C, Marbaix E, Henriet P (1824). Regulation of matrix metalloproteinases activity studied in human endometrium as a paradigm of cyclic tissue breakdown and regeneration. Biochim Biophys Acta.

[7] Nagase H, Murphy G, Edwards D, Hoyer-Hansen G, Blasi F, Sloane BF (2009). Tailoring TIMPs for selective metalloproteinase inhibition. The cancer degradome.

[8] Brew K, Nagase H (1803). The tissue inhibitors of metalloproteinases (TIMPs): an ancient family with structural and functional diversity. Biochim Biophys Acta.

[9] Laflamme BA, Wolfner MF (2013). Identification and function of proteolysis regulators in seminal fluid. Mol Reprod Dev.

[10] Shin SB, Cho JW, Lee SH, Yang KM, Lim CK, Lee HS (2013). Fertilization and pregnancy potential of immature oocytes from stimulated intracytoplasmic sperm injection cycles. Clin Exp Reprod Med.

[11] Gardner DK, Schoolcraft WB, Jansen R, Mortimer D (1999). In vitro culture of human blastocysts. Toward reproductive certainty: fertility and gentics beyond.

[12] Rosen MP, Zamah AM, Shen S, Dobson AT, McCulloch CE, Rinaudo PF (2009). The effect of follicular fluid hormones on oocyte recovery after ovarian stimulation: FSH level predicts oocyte recovery. Reprod Biol Endocrinol.

[13] Lee DM, Lee TK, Song HB, Kim CH (2005). The expression of matrix metalloproteinase-9 in human follicular fluid is associated with in vitro fertilisation pregnancy. BJOG.

[14] Hsieh WY, Kuan TC, Cheng KS, Liao YC, Chen MY, Lin PH (2012). ACE/ACE2 ratio and MMP-9 activity as potential biomarkers in tuberculous pleural effusions. Int J Biol Sci.

[15] Cheng KS, Liao YC, Chen MY, Kuan TC, Hong YH, Ko L (2013). Circulating matrix metalloproteinase-2 and -9 enzyme activities in the children with ventricular septal defect. Int J Biol Sci.

[16] Hu X, Beeton C (2010). Detection of functional matrix metalloproteinases by zymography. J Vis Exp.

[17] Sessions DR, Vick MM, Fitzgerald BP (2009). Characterization of matrix metalloproteinase-2 and matrix metalloproteinase-9 and their inhibitors in equine granulosa cells in vivo and in vitro. J Anim Sci.

[18] McCaffery FH, Leask R, Riley SC, Telfer EE (2000). Culture of bovine preantral follicles in a serum-free system: markers for assessment of growth and development. Biol Reprod.

[19] Horka P, Malickova K, Jarosova R, Janatkova I, Zima T, Kalousova M (2012). Matrix metalloproteinases in serum and the follicular fluid of women treated by in vitro fertilization. J Assist Reprod Genet.

[20] Grabiec M, Szymański W, Szymański M, Jendryczka J, Polak G, Gogacz M (2001). Concentrations of MMP-1, TIMP-1, MMP-1/TIMP-1 and I CTP complexes in follicular fluid as related to fertilization rate in women treated with in-vitro fertilization. Ginekol Pol.

[21] Bilen E, Tola EN, Oral B, Doguç DK, Günyeli I, Köse SA, Ilhan I. Do follicular fluid gelatinase levels affect fertilization rates and oocyte quality? Arch Gynecol Obstet. 2014;290:1265-71. 10.1007/s00404-014-3370-x 25027821

[22] Hulboy DL, Rudolph LA, Matrisian LM (1997). Matrix metalloproteinases as mediators of reproductive function. Mol Hum Reprod.

[23] Riley SC, Gibson AH, Leask R, Mauchline DJ, Pedersen HG, Watson ED (2001). Secretion of matrix metalloproteinases 2 and 9 and tissue inhibitor of metalloproteinases into follicular fluid during follicle development in equine ovaries. Reproduction.

[24] Jamnongjit M, Gill A, Hammes SR (2005). Epidermal growth factor receptor signalling is required for normal ovarian steroidogenesis and oocyte maturation. Proc Natl Acad Sci U S A.

[25] Panigone S, Hsieh M, Fu M, Persani L, Conti M (2008). Luteinizing hormone signaling in preovulatory follicles involves early activation of the epidermal growth factor receptor pathway. Mol Endocrinol.

[26] Nyholt de Prada JK N, Lee YS, Latham KE, Chaffin CL, VandeVoort CA. Role for cumulus cell-produced EGF-like ligands during primate oocyte maturation in vitro. Am J Physiol Endocrinol Metab. 2009;296:E1049–58. 10.1152/ajpendo.90930.2008 PMC2681310 19276391

[27] Shiraishi K, Ascoli M (2008). A co-culture system reveals the involvement of intercellular pathways as mediators of the lutropin receptor (LHR)-stimulated ERK1/2 phosphorylation in Leydig cells. Exp Cell Res.

[28] Carbajal L, Biswas A, Niswander LM, Prizant H, Hammes SR (2011). GPCR/EGFR cross talk is conserved in gonadal and adrenal steroidogenesis but is uniquely regulated by matrix metalloproteinases 2 and 9 in the ovary. Mol Endocrinol.

[29] Ashkenazi H, Cao X, Motola S, Popliker M, Conti M, Tsafriri A (2005). Epidermal growth factor family members: endogenous mediators of the ovulatory response. Endocrinology.

[30] Rispoli LA, Payton RR, Gondro C, Saxton AM, Nagle KA, Jenkins BW (2013). Heat stress effects on the cumulus cells surrounding the bovine oocyte during maturation: altered matrix metallopeptidase 9 and progesterone production. Reproduction.

[31] Ferrer M, Rodriguez H, Zara L, Yu Y, Xu W, Oko R (2012). MMP2 and acrosin are major proteinases associated with the inner acrosomal membrane and may cooperate in sperm penetration of the zona pellucida during fertilization. Cell Tissue Res.

[32] Iwao Y, Shiga K, Shiroshita A, Yoshikawa T, Sakiie M, Ueno T (2014). The need of MMP-2 on the sperm surface for Xenopus fertilization: Its role in a fast electrical block to polyspermy. Mech Dev.

[33] Arslan M, Morshedi M, Arslan EO, Taylor S, Kanik A, Duran HE (2006). Predictive value of the hemizona assay for pregnancy outcome in patients undergoing controlled ovarian hyperstimulation with intrauterine insemination. Fertil Steril.

